# Eco-evolutionary Model of Rapid Phenotypic Diversification in Species-Rich Communities

**DOI:** 10.1371/journal.pcbi.1005139

**Published:** 2016-10-13

**Authors:** Paula Villa Martín, Jorge Hidalgo, Rafael Rubio de Casas, Miguel A. Muñoz

**Affiliations:** 1 Departamento de Electromagnetismo y Física de la Materia and Instituto Carlos I de Física Teórica y Computacional, Universidad de Granada, Granada, Spain; 2 Dipartimento di Fisica ’G.Galilei’ and CNISM, INFN, Università di Padova, Padova, Italy; 3 Estación Experimental de Zonas Áridas, EEZA-CSIC, Almería, Spain; 4 UMR 5175 Centre d’Ecologie Fonctionnelle et Evolutive (CNRS), Montpellier, France; 5 Departamento de Ecología, Universidad de Granada, Granada, Spain; University of Chicago, UNITED STATES

## Abstract

Evolutionary and ecosystem dynamics are often treated as different processes –operating at separate timescales– even if evidence reveals that rapid evolutionary changes can feed back into ecological interactions. A recent long-term field experiment has explicitly shown that communities of competing plant species can experience very fast phenotypic diversification, and that this gives rise to enhanced complementarity in resource exploitation and to enlarged ecosystem-level productivity. Here, we build on progress made in recent years in the integration of eco-evolutionary dynamics, and present a computational approach aimed at describing these empirical findings in detail. In particular we model a community of organisms of different but similar species evolving in time through mechanisms of birth, competition, sexual reproduction, descent with modification, and death. Based on simple rules, this model provides a rationalization for the emergence of rapid phenotypic diversification in species-rich communities. Furthermore, it also leads to non-trivial predictions about long-term phenotypic change and ecological interactions. Our results illustrate that the presence of highly specialized, non-competing species leads to very stable communities and reveals that phenotypically equivalent species occupying the same niche may emerge and coexist for very long times. Thus, the framework presented here provides a simple approach –complementing existing theories, but specifically devised to account for the specificities of the recent empirical findings for plant communities– to explain the collective emergence of diversification at a community level, and paves the way to further scrutinize the intimate entanglement of ecological and evolutionary processes, especially in species-rich communities.

## Author Summary

Population ecology and evolutionary biology have been traditionally studied as separate disciplines, even if feedbacks between community and evolutionary processes are known to exist, having been empirically characterized in recent years in different types of communities (from microbes to plants and vertebrates), and theoretically analyzed with novel and powerful mathematical tools. Recent long-term field experiments with plants have proven that rapid co-evolution and diversification of species traits results in an overall enhancement of the ecosystem productivity, with important consequences for agriculture and conservation. Here, we propose a relatively simple computational eco-evolutionary model specifically devised to describe rapid phenotypic diversification in this type of species-rich communities. Our model captures the main phenomenology observed experimentally, and it also makes non-trivial predictions for long term phenotypic change and ecological interactions, such as the stable coexistence of highly specialized species or the possible emergence of phenotypically equivalent species occupying the same niche. Finally, the model is easily generalizable to analyze different eco-evolutionary problems within a relatively simple and unified computational framework.

## Introduction

Community ecology studies how the relationships among species and their environments affect biological diversity and its distribution, usually neglecting phenotypic, genetic and evolutionary changes [1–3]. In contrast, evolutionary biology focuses on genetic shifts, variation, differentiation, and selection, but –even if ecological interactions are well-recognized to profoundly affect evolution [4]– community processes are often neglected. Despite this apparent dichotomy, laboratory analyses of microbial communities and microcosms [5–14] as well as long-term field experiments with plant communities [15, 16] and vertebrates [17, 18] provide evidence that species can rapidly (co)evolve and that eco- and evolutionary processes can be deeply intertwined even over relatively short (i.e. observable by individual researchers) timescales [19].

Over the last two decades or so, the need to consider feedbacks between ecological and evolutionary processes has led many authors to develop a framework to merge together the two fields [20–41]. In particular, the development of quantitative trait models [42] and the theories of adaptive dynamics [43, 44] and adaptive diversification [22–24, 26–28, 34], reviewed in [40, 42], has largely contributed to the rationalization of eco-evolutionary dynamics, shedding light onto non-trivial phenomena such as sympatric speciation and evolutionary branching [40].

**Fig 1:**
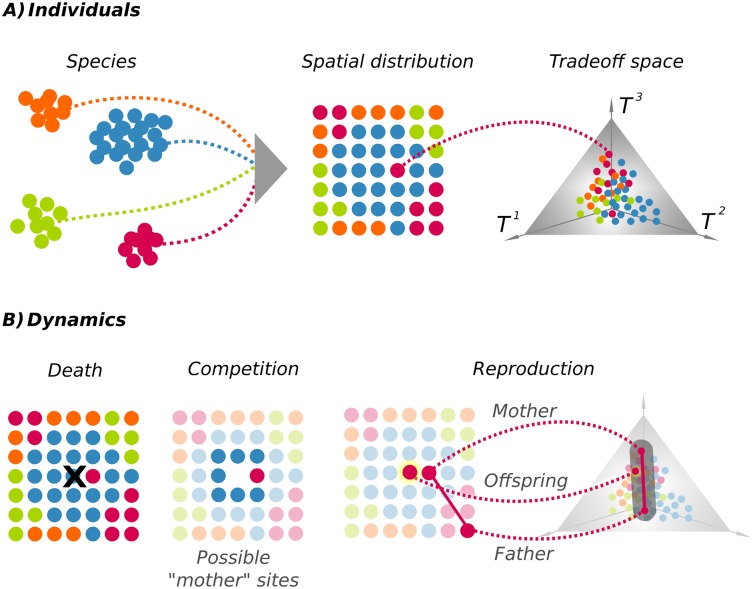
(Color online) Sketch of the model. (**A**) Individuals of different species (different colors) compete for available resources in a physical space (two-dimensional square lattice), which is assumed to be saturated at all times. Each individual is equipped with a set of phenotypic traits that corresponds to a single point in the trade-off space. This is represented here (as a specific example) as an equilateral triangle (a “simplex” in mathematical terms) corresponding to the case of 3 coordinates which add up to 1 (e.g., fraction of the total biomass devoted to roots, leaves/stems and flowers, respectively [41]). For instance, a point close to vertex *T*^1^ exploits better the limiting resource 1 (e.g. soil nutrients) than another one near vertex *T*^2^, but is less efficient at exploiting resource 2 (e.g. light) than this latter one (see Methods). (**B**) Individuals die after one timestep, giving rise to empty sites; each of these is occupied by an offspring from a “mother” within its local neighborhood (consisting of 8 sites in the sketch for clarity, although we considered also a second shell of neighbors in the simulations, i.e. a kernel of 24 sites). The mother is randomly selected from the plants occupying this neighborhood in the previous generation, with a probability that decreases with the level of similarity/competition with its neighbors (see Methods). The implanted seed is assumed to have been fertilized by a conspecific “father” from any arbitrary random location, selected also with a competition-level dependent probability. The offspring inherits its phenotype from both parents; its traits can lie at any point (in the shaded region of the figure) nearby the the parental ones, allowing for some variation. For a given number of initial species *S*, two key parameters control the final outcome of the dynamics: *β*, characterizing the overall level of competition, and *μ*, representing the variability of inherited traits. We fix most of the parameters in the model (lattice size, individuals within the competition/reproduction kernel, etc.) and study the dependence on *S*, *β* and *μ*.

On the empirical side, the recent work by Zuppinger-Dingley *et al*. on long-term field experiments of vegetation dynamics appears to confirm many of the theoretical and observational predictions [45]. This study provided strong evidence for the emergence of *rapid collective evolutionary changes*, resulting from the selection for complementary character displacement and niche diversification, reducing the overall level of competition and significantly increasing the ecosystem productivity within a relatively short time. This result is not only important for understanding rapid collective evolution, but also for designing more efficient agricultural and preservation strategies. More specifically, in the experimental setup of Zuppinger-Dingley and colleagues, 12 plant species of different functional groups were grown for 8 years under field conditions either as monocultures or as part of biodiverse communities. Collecting plants (seedlings and cutlings) from these fields, propagating them in the laboratory, and assembling their offspring in new communities, it was possible to quantify the differences between laboratory mixtures consisting of plants with a history of isolation (i.e. from monocultures) and plants from biodiverse fields. While the former maintained essentially their original phenotypes, the latter turned out to experience significant complementary trait shifts –e.g. in plant height, leaf thickness, etc.– which are strongly suggestive of a selection for phenotypic and niche differentiation [41] (see Fig 1 therein). Furthermore, there were strong *net biodiversity effects* [46], meaning that the relative increase in total biomass production in laboratory mixtures with respect to laboratory monocultures was greater for plants from biodiverse plots than for plants coming from monocultures. These empirical results underscore the need for simple theoretical methodologies, in the spirit of the above-mentioned synthetic approaches [20, 23, 27, 28, 36, 40, 42, 47, 48]. These approaches should explain the community and evolutionary dynamics of complex and structured communities such as the ones analyzed in [45].

The phenotypic differentiation observed in the experiments of Zuppinger-Dingley et al. might be partially rationalized within the framework of relatively simple deterministic approaches to eco-evolution such as adaptive dynamics (see e.g. [20, 23, 27, 28, 36, 40, 42, 47]). In this context, diversification is the natural outcome of an adaptive/evolutionary process that increases fitness by decreasing competition through trait divergence.

However, it is not obvious what would be the combined effects in this simplistic version of adaptive dynamics of introducing elements such as sexual reproduction, space, and multi-species interactions that could play an important role in shaping empirical observations. Moreover, questions such as whether phenotypic differentiation occurs both above and below the species level (i.e., within species or just between them), the possibility of long term coexistence of phenotypically equivalent species in the presence of strong competition (i.e., emergent neutrality), or the expected number of generations needed to observe significant evolutionary change remain unanswered and require a more detailed and specific modeling approach, within the framework of adaptive dynamics.

Thus, our aim here is to contribute to the understanding of eco-evolutionary dynamics, emphasizing collective co-evolutionary aspects rather than focusing on individual species or pairs of them. For this purpose, we developed a simple computational framework –similar to existing approaches (see Discussion)– specifically devised at understanding the emerging phenomenology of the experiments of Zuppinger-Dingley *et al*. In particular, we propose an individual-based model, with spatial structure, stochasticity, sexual reproduction, mutation, multidimensional trait-dependent competition and, importantly, more than-two-species communities (in particular, possibly owing to analytical difficulties, relatively limited work has been published about more than three-species communities, which is crucial to achieve a realistic integration of ecological and evolutionary dynamics for natural communities; see however [49–51]). Furthermore, our method is flexible enough as to be easily generalizable to other specific situations beyond plant communities and can rationalize the circumstances under which phenotypic diversification and niche specialization may emerge using simple, straightforward rules.

## Results

### Model essentials

We construct a simple model which relies on both *niche* based approaches [52–54] and *neutral* theories [55–58]. The former prioritize trait differences and asymmetric competition, underscoring that coexisting species must differ in their eco-evolutionary trade-offs, i.e., in the way they exploit diverse limiting resources, respond to environmental changes, etc., with each trade-off or “niche” choice implying superiority under some conditions and inferiority under others [1, 3, 53, 54]. Conversely, neutral theory ignores such asymmetric interactions by making the radical assumption of species equivalence, and focuses on the effects of demographic processes such as birth, death and migration.

Here, we adopt the view shared by various authors [36, 59–61] that niche-based and neutral theories are complementary extreme views. In what follows, we present a simple model that requires of both neutral and niche-based elements. In particular, our model incorporates trade-off-based features such as the existence of heritable phenotypic traits that characterize each single individual. However, the impact of these traits on individual fitness is controlled by a model parameter, that can be tuned to make the process more or less dependent on competition, in the limit even mimicking neutral (or “symmetric”) theories [55, 56].

The traits of each single individual are determined by quantitative phenotypic values that can be regarded as the investment in specific functional organs. For instance, the traits could represent the proportion of biomass devoted to exploit soil nutrients (roots), light (leaves and stems), and to attract pollinators and capture pollen (flowers; see Fig 1). We then assume a hard limit –constant across generations– to the amount of resources that can be devoted to generate the phenotype, i.e. it is impossible to increase all phenotypic values simultaneously. Thus each individual is constrained to make specific trade-offs in the way it exploits resources. Because similar values in the trade-off space entail comparable exploitation of the same resource (e.g., water, light or pollinators) similar individuals experience higher levels of competition, which translates into a lower fitness. This can be regarded as a frequency dependent selection mechanism providing an adaptive advantage to exceptional individuals, able to exploit available resources. Therefore, the ecological processes of competition, reproduction, and selection lead to evolutionary shifts in the distribution of phenotypic traits which feed back into community processes, giving rise to integrated eco-evolutionary dynamics.

### Model construction

The basic components of the model are as follows (further details are deferred to the Methods section). We consider a community of individuals of *S* different species, that are determined initially by mating barriers (i.e. a species is defined as a set of individuals that can produce fertile offspring [62]). Each individual occupies a position in physical space (represented as a saturated square lattice) and is characterized by the label of the species to which it belongs and a set of intrinsic parameters (i.e. trait values), specifying its coordinates in the “trade-off space” as sketched in Fig 1 (see also [41, 63]. All positions within the trade-off space are assumed to be equally favorable *a priori*. In what follows, we make a perfect identification between the trade-offs of a given individual and its phenotypic traits, which also determine the “niche” occupied by each individual. In principle, each individual, regardless of its species, can occupy any positition in the trade-off space. Positions near the center of the trade-off space (Fig 1) correspond to phenotypes with similar use of the different resources (i.e., “generalists”), while individuals near the corners specialize in the exploitation of a given resource (“specialists”).

Individuals are subjected to the processes of birth, competition for resources, reproduction, descent with modification, and death. Individuals are assumed to undergo sexual reproduction, as in the experiments of [45] (implementations with asexual reproduction are discussed later); they are considered to be semelparous, so that after one simulation time step (i.e, a reproductive cycle) they all die and are replaced by a new generation. Importantly, demographic processes are strongly dependent on phenotypic values. In particular, the main niche-based hypothesis is that individual organisms with a better “performance” are more likely to reproduce than poorly performing ones. To quantify the notion of “performance”, we rely on classical concepts such as limiting similarity, competitive exclusion principle and niche overlap hypothesis [64, 65], which posit that in order to avoid competition, similar species must differ in their phenotypes. More specifically, our model assumes that the performance of a given individual increases with its trait “complementarity” to its spatial neighbors [65], as quantified by its averaged distance to them in trade-off space (see Methods); i.e. the larger the phenotypic similarity among neighbors, the stronger the competition, and the worse their performance. Although the performance of a given individual depends on its complementarity with its neighbors, the model is symmetric among species and phenotypes; performance is blind to species labels and does not depend on the specific location in the trade-off space.

The reproduction probability or performance of any given individual is mediated by a parameter *β* which characterizes the global level of competitive stress in the environment (see Methods). In the limit of no competition, *β* = 0, the dynamics become blind to phenotypic values and can be regarded as fully neutral, while in the opposite limit of extremely competitive environments, *β* → ∞, niche effects are maximal and a relatively small enhancement of trait complementarity induces a huge competitive advantage. Finally, a mother selected as described in the competition process is assumed to be fertilized by a conspecific “father” in the population (interspecies hybridization is not considered here) which is also selected with the same reproduction probability function based on its performance. The offspring inherits its traits from both parents, with admixture and some degree of variation *μ* (see Fig 1 and Methods). This process is iterated for all lattice sites and for an arbitrarily large number of reproductive cycles, resulting in a redistribution of species both in physical and in trade-off space. Species can possibly go extinct as a consequence of the dynamics. In this version of the model, speciation is not considered, though it could be easily implemented by establishing a dependence of mating on phenotypic similarity, making reproduction between sufficiently different individuals impossible [37].

### Computational results

**Fig 2:**
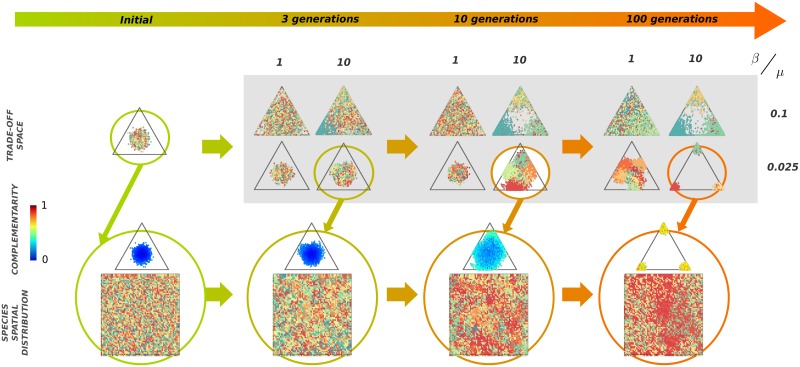
(Color online) Illustration of the emergence of rapid phenotypic diversification for a computational system of size 64 × 64 and 16 species (labeled with different colors). (**Top**).**Phenotypic diagrams** measured at different evolution stages (1, 3, 10 and 100 generations, respectively) for different values of the two parameters: level of competition *β* (1 for the case of low competition and 10 for strong competition) and variation in inherited traits *μ* (0.1 for large variation and 0.025 for small variation). In all cases, phenotypic differentiation among species is evident even after only 10 generations. In the long term (100 generations) species diversification and specialization is most evident for small *μ* and large *β*; in this last case, different species (colors) can coexist for large times in the same region/corner of trade-off space. (**Central**). **Complementarity diagrams** representing the values of averaged local complementarity for all individuals of any species for small *μ* (0.025) and large *β* (10). Individuals with small complementarity (i.e. under strong competition with neighbors) disappear in the evolutionary process, while communities with high degrees of local complementarity are rapidly selected. (**Bottom**). **Spatial distribution of species** for different number of generations. As a result of the eco-evolutionary dynamics, anti-correlated patterns –in which neighboring plants tend to be different– emerge (note that colors represent species assignment and do not reflect phenotypic values).

**Fig 3:**
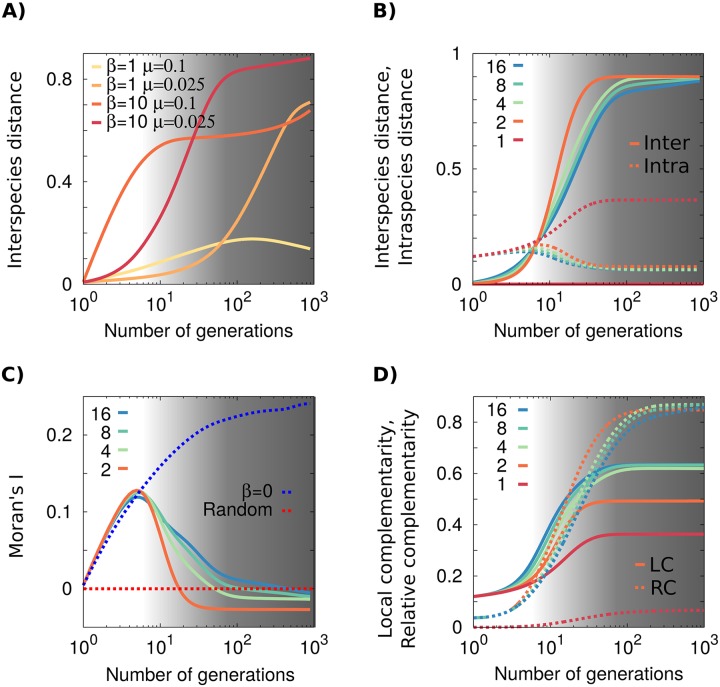
(Color online) Measurements of different biodiversity indices. (**A**)**Phenotypic distances among species** grow systematically during the eco-evolutionary process, reflecting a clear tendency towards species differentiation (same sets of parameter values as in Fig 2, *S* = 16). Differentiation is faster for relatively small values of trait variability *μ* and large values of the competitive stress *β*. (**B**) **Phenotypic differentiation among and within species**. While interspecies distances grow in time for all values of *S* and converge to similar values on the long term, intraspecific phenotypic variability is much larger on the long term for monocultures than for biodiverse mixtures. (**C**) **Phenotypic similarity among close neighbors**. Moran’s index (*I*) for *β* = 10 and different values of *S* as well as for *β* = 0 and for a random distribution (i.e. in the absence of spatial interactions). The value of *I* tends to 0 for random distributions, is positive for *β* = 0, and tends to small negative values for *β* ≠ 0. Whenever competition depends on the phenotypic values (i.e., *β* > 0) the system avoids close cohabitation of individuals of the same species. This negative spatial autocorrelation results in *I* < 0; in all cases, *μ* = 0.025. (**D**) **Averaged local and relative complementarity** in the community increase with time and reach larger values for more biodiverse communities. The phenotypic differentiation among individuals is greater both among close neighbors and at the global scale as the number os species *S* increases. In all plots, parameters are *L* = 64 and, unless it is specified, *β* = 10 and *μ* = 0.025; curves are averaged over at least 10^3^ runs; shaded light grey areas stand for times during which extinction tends to occur causing *S* to decrease (see S10 Appendix in S1 Text for details), while in dark grey ones the system tend to stabilize at a given final number of species.

Simulations are started with individuals of *S* different species (e.g. *S* = 16) randomly distributed in space. In the initial conditions, the traits of all individuals are a sample from a common Gaussian distribution centered around the center of the simplex (note that as shown in the S5 Appendix in S1 Text, results do not depend on the particular choice of initial conditions). Statistical patterns emerging from the eco-evolutionary dynamics described above are analyzed as a function of the number of generations and as a function of the number of species *S*, for different values of the two free parameters: the overall level of competition *β* and the variability of inherited traits *μ*. Results are illustrated in Fig 2 showing (*i*) phenotypic diagrams (top row) specifying the position of each single individual and its species in the trade-off space for different parameter values and evolutionary times (*ii*); values of complementarity for all individuals (central row) in the trade-off space, and (*iii*) the spatial distribution of individuals and species (bottom row). Finally, several biodiversity indices are reported in Fig 3.

#### Species differentiation

As illustrated in Fig 2 (shaded area), different distributions of individuals in the trade-off space appear depending on the specific values of *β* and *μ*. Visual inspection reveals the emergence of rapid phenotypic differentiation, i.e. segregation of colors in trade-off space after a few (e.g. 10) reproductive cycles. The segregation is much more pronounced for relatively small variability (e.g. *μ* = 0.025) and large competitive stress (e.g. *β* = 10). This is quantified (see Fig 3A) by the average interspecies distance (see Methods), whose specific shape depends on parameter values. As shown in Fig 3B, the fastest growth is obtained for *S* = 2, but the curves converge to a constant value (mostly independent of *S*) after a sufficiently large number of generations. Moreover, as shown in the central row of Fig 2 the complementarity –averaged over all individuals in the community (see Methods)– also grows during the course of evolution (i.e. colors shift from blue to yellowish). Observe in Fig 2 that, for asymptotically large evolutionary times, there is a tendency for all species to cluster around the corners of the trade-off space, suggesting that the optimal solution to the problem of minimizing the competition with neighbors corresponds to communities with highly specialized species. This specialization does not occur in monocultures (*S* = 1), as sexual mating pulls the species together and avoids significant phenotypic segregation.

#### Emergence of local anti-correlations

The high level of phenotypic specialization observed after large evolutionary times for large competition stress and small variability, might seem in contradiction with the overall tendency to niche differentiation. In other words, most of the trade-off space becomes empty in this case, while individuals aggregate at the (highly populated) corners. The answer to this apparent conundrum is that similarly specialized individuals have a statistical tendency to avoid being spatial neighbors. Indeed, as qualitatively illustrated in the lowest right panel of Fig 2, extreme specialization is accompanied by a tendency to diminish spatial clustering, i.e. to create spatial anti-correlations within each species. This tendency –which stems from intraspecific competition and opposes to the demographic tendency of similar individual to cluster in space– is quantitatively reflected by negative values of Moran’s index *I* (see Fig 3C and Methods). Note also that *I* and thus the spacial distribution of species, is radically different in the presence and in the absence of competition (i.e. for *β* ≠ 0 and *β* = 0, respectively) as can be seen in Fig 3C. In the absence of competition, species are distributed randomly forming aggregated spatial clusters without competition-induced local anti-correlations.

#### Intraspecific diversity

This quantity is defined as the mean “complementarity” among all pairs of conspecific individuals in the community, and illustrates the level of phenotypic diversity within species. As shown in Fig 3B, the intraspecific diversity is much larger for monocultures. In monocultures, neighbors are obviously conspecific and the only available mechanism to reduce overall competition is to increase intraspecific diversity. Therefore, as a general result, monocultures tend to enhance their intraspecific phenotypic distances, while biodiverse communities tend to enhance phenotypic differentiation among species but result in more similar conspecifics.

#### Local complementarity

Fig 3D shows the evolution of the mean complementarity of individuals respect to its spatial neighbors. This averaged *local* complementarity (LC) controls the dynamics and the actual reduction in the level of competition for a given spatial distribution, and is much larger for mixtures than for monocultures (it grows monotonously with *S* and saturates at a maximal value).

#### Global complementarity

Similarly, we can measure “*global*” complementarity (GC), i.e. the average phenotypic distance among all individuals in the experiment, regardless of their spatial coordinates, after a given number of generations. Additionally, we measured GC_intra_ (resp. GC_inter_) which is GC averaged only over individuals of the same (resp. different) species (see Methods). In Fig 3D we present results for the *relative* complementarity RC = GC_inter_ − GC_intra_, which is a measure of the averaged difference in the level of competition between randomly sampled non-conspecific and conspecific individuals, respectively. Observe that the RC is larger for mixtures than for monocultures, RC(*S* > 1) > RC(*S* = 1), and that it grows faster in time for smaller values of *S* (e.g. *S* = 2), but reaches almost equal constant values after a sufficiently large number of generations.

#### Emergent neutrality

As illustrated in Fig 2, different species with very similar trait values can coexist (e.g. yellow and orange species at the right corner of the phenotypic diagram for *μ* = 0.025 and *β* = 10 in Fig 2) even after many generations. Such a coexistence emerges spontaneously and although it is transitory it can last for arbitrarily long times provided that the system size is sufficiently large. From an ecological point of view, these species can be regarded as functionally equivalent as they occupy the same niche region (see S6 Appendix in the S1 Text for a detailed analysis of the stability and coexistence time of such species).

#### Model variants

To investigate the generality of our findings, we also explored whether the main conclusions are robust against some constraints of the implementation. We briefly explain the variants we took into account below. i) *Non-symmetrical phenotypic trade-offs*: as a first step, we assumed that not all positions in the trade-off space are equally rewarding *a priori*: individuals in certain regions of the trade-off space have larger reproduction probabilities than others. Non-symmetrical trade-offs lead to very similar results as above, confirming the robustness of our conclusions (see S7 Appendix in S1 Text). ii) *Asexual reproduction*: as shown in S8 Appendix in S1 Text, for communities of individuals able to reproduce asexually (i.e. assuming transmission of traits only from the mother) the outcome of the model is different: individuals tend to diversify, but such diversification occurs even within species (i.e. intra-specific diversification is much larger than in the sexual case); in other words, since there is no admixing of the phenotypic traits through reproduction, diversification occurs within maternal lineages, rather than at the species level. iii) *Long-distance dispersal and competition*: we also studied the case in which dispersal and competition are long-distance processes and affect the whole community and not only close neighbors; as shown in S3 Appendix in S1 Text the phenomenology reported above remains quite similar, even if in this well-mixed case the co-existence of phenotypically-equivalent species is less likely (owing to the lack of spatial separation). iv) *Effect of the competition kernel*: the specific form of the competition kernel can play a crucial role in the formation of species clusters in phenotypic space [66–72]; in the S9 Appendix of the S1 Text we explore different kernel functions and show that our results are robust against changes in the mathematical expression of competition.

## Discussion

In the present paper, we have developed a parsimonious modeling approach to integrate important ecological and evolutionary processes. In particular, we focused on understanding rapid phenotypic diversification observed in complex biological communities of plants such as those recently reported by Zuppinger-Dingley *et al*. in long-term field experiments [41, 45].

Our model blends standard community processes, such as reproduction, competition or death, with evolutionary change (e.g., descent with modification); i.e. community and evolutionary dynamics are coupled together, feeding back into each other. Over the last decades, attempts to integrate ecological and evolutionary dynamics have been the goal of many studies (see e.g. [16, 22–34, 37, 39, 40, 48, 63]). In particular, a basic algorithm for modeling eco-evolutionary dynamics as a stochastic process of birth with mutation, interaction, and death was proposed in [22] and much work has been developed afterwards to incorporate elements such as spatial effects and different types of interspecies interactions [28].

Rather than providing a radically different framework, our model constitutes a blend of other modeling approaches in the literature of eco-evolutionary processes, and in fact it shares many ingredients with other precedent works, specially with the theory of adaptive dynamics [40, 42]. For instance, Gravel et al. [36] also considered a spatially-explicit individual-based model with trait-dependent competition. However, our work has been specifically devised to shed light on the experimental findings of Zuppinger et al. [45], and puts the emphasis on communities with arbitrarily large number of species, while usually the focus is on the (co-)evolution of pairs of species (e.g. predator-prey, host-parasite, etc.) or speciation/radiation of individual species. Finally, our modelling approach is sufficiently general as to be flexible to be adapted to other situations with slightly different ingredients. We explored some of these possible extensions in some Appendices (S3,S4,S7,S8,S9) in S1 Text (e.g long-distance dispersal, asexual reproduction, etc.), but other studies can be built upon the work laid here in a relatively simple way.

The present model relies on a number of specific assumptions, two of which are essential in that they couple community and evolutionary dynamics: i.e. (i) demographic processes are controlled by competition for resources which is mediated by phenotypic traits and (ii) successful individuals are more likely to transmit their phenotypes to the next generation with some degree of variation. These two ingredients are critical for the emerging phenomenology. For instance, in the absence of competition (i.e. *β* = 0) reproduction probabilities are identical for all individuals, implying that the model becomes neutral, and the evolutionary force leading to species differentiation vanishes (see S4 Appendices in S1 Text). On the other hand, variation in inherited traits is necessary to allow for the emergence of slightly different new phenotypes and the emergence of drifts in trade-off-space. Although these constraints might be regarded as limiting, we deem them biologically realistic and do not think they hamper the predictive power of our model. Most of the remaining ingredients, such as the existence of a saturated landscape, semelparity (i.e. non-overlapping generations), the specific form in which we implemented initial conditions, competition, dispersion, selection, inheritance linked to phenotypic characters rather than to a genotypic codification, etc. can be modified without substantially affecting the results. This flexibility could make the description of other type of communities possible with minimal model variations. Similarly, the model could be extended to incorporate phenotype-dependent reproductive barriers (and thus speciation) and the possibility of interspecies hybridization by making reproduction a function of phenotypic distance and relaxing its dependency on species labels.

In addition to rapid phenotypic diversification, the experiments of Zuppinger et al. found an enhancement of the overall productivity in mixtures of diverse plants with respect to monocultures of the same plants [45]. Our model cannot be used to directly quantify such “biodiversity effects” [46], as we assume a fully saturated landscape and there is no variable that accounts for total biomass production. However, in principle, under the hypothesis that larger trait complementarities correlate with greater resource capture and biomass production, the observed increase of relative complementarity in mixtures (see Fig 3) could be used as a proxy for biodiversity effects. Observe, nonetheless, that the previous assumption might by wrong (or incomplete) as productivity can be profoundly affected by other factors such as, for instance, positive interactions between similar species, not modeled here, and more sophisticated approaches –see [73–77]– are necessary to validate this hypothesis. In the future we plan to modify our model to represent non-saturated landscapes and more detailed ecological dynamics, allowing for explicit analyses of biodiversity-productivity relationships.

Beyond explaining most of the empirical observations in [45], our model leads to some far-reaching predictions (some of them already shared by existing theories); one of the most remarkable ones is that optimal exploitation of resources comes about when the full community evolves into a reduced number of highly specialized species –the exact number depending on the dimensions of the trade-off space– that coexist in highly dispersed and intermixed populations. Such specialization might be unrealistic in the case in which all traits in trade-off space are essential for survival, and thus the convergence toward perfect specialization is capped. In any case, this result is congruent with the niche dimension hypothesis [78], that postulates that a greater diversity of niches entails a greater diversity of species, i.e. a larger number of limiting factors (and thus of possible trade-offs) leads to richer communities [79]. However, this outcome might be affected by perturbations (migration, environmental variability, etc) which could be easily implemented in our model, and could prevent real communities from reaching the asymptotic steady state predicted here. It is also noteworthy that the resulting highly specialized species can be phenotypically equivalent, and a set of them can occupy almost identical locations in the trade-off space. Such species equivalence appears spontaneously, and supports the views expressed by other authors that “emergent neutrality” is a property of many ecosystems [80–82]. In future work we will explore the possibility of phase transitions separating an ecological regime based on the coexistence of multiple highly specialized species from an ecosystem dominated by generalists and the conditions under which each regime emerges.

Beyond phenotype-dependent mating, upcoming studies will extend our approach to address communities where collective diversification phenomena based on both competition and cooperation are known to emerge (see e.g. [13]), as well as investigate the evolution of communities with distinct types of interacting species such as plant-pollinator mutualistic networks. This research will hopefully complement the existing literature and help highlighting the universal and entangled nature of eco-evolutionary processes.

## Methods

### Model implementation

We implemented computer simulations in which each individual plant, *i*, is fully characterized by (see also Fig 1): (*i*) a label identifying its species, (*ii*) its coordinates in the physical space, and (*iii*) a set of real numbers specifying its phenotypic traits. In these simulations, time can be implemented either as discrete/synchronous updating or continuous/sequential updating without significantly altering the results. *Species–*, we consider a fixed number of species, labeled from 1 to *S*; while the emergence of new species is not considered here, some of them may become extinct along the course of evolution. *Physical space–* We consider a two-dimensional homogeneous physical space described by a *L* × *L* square lattice, assumed to be saturated at all times, in which the neighborhood of each individuals is determined by the closest *K* sites (in our simulations, we took *L* = 64 and *K* = 24). *Phenotypic traits and trade-off space–* As energy and resources are limited, each individual plant needs to make specific choices/trade-offs on how to allocate different functions. The way we implement the “trade-off space” is inspired in the field of multi-constraint (non-parametric) optimization that it is called Pareto optimal front/surface [83]; it includes the set of possible solutions such that none of the functions can be improved without degrading some other. Thus, the phenotype of any individual can be represented as a trade-off equilibrium, a point in this space and encapsulated in a set of real numbers **T** = (*T*^1^, *T*^2^, …, *T*^*n*^) (all of them in the interval [0, 1]), such that $\sum_{k=1}^{n}T^{k}=1$ where *n* is the number of trade-offs (see Fig 1 and [41]). All positions within the trade-off space are equivalent a priori, although this requirement can be relaxed. *Competition for resources*– The trait “complementarity” between two individuals *i* and *j* is quantified as their distance in the trade-off space: $c_{ij}=\sum_{k=1}^{n}\left|T^{k}\left(i\right)-T^{k}\left(j\right)\right|/n$, which does not depend on species labels. The averaged complementarity, (or simply “complementarity”) over all the neighbors *j* of individual *i* is *C*_*i*_ = ∑_*j* ∈ *n*.*n*.(*i*)_ *c*_*ij*_/*K*. *Complementarity-based dynamics–* Each timestep, every individual is removed from the population; the resulting vacant site *i* is replaced by an offspring of a potential mother plant *j* which is selected from the list of *K* local neighbors of the vacant site with a given probability *P*_mother_(*j*). This probability controls the dynamical process; we assume it to increase as the mother’s trait complementarity *C*_*j*_ increases (i.e. as its effective competitive stress diminishes): $P_{\text{mother}}\left(j\right)=e^{\beta C_{j}}/\sum_{j^{\prime }\in n.n.\left(i\right)}e^{\beta C_{j^{\prime }}}\;$, where the sum runs over the set of *K* neighbors of *i*; *e*^*βC*_*j*_^ is the “performance” of individual *j* and *β* is a tunable “competition parameter” controlling the overall level of competitive stress in the community. Once the mother has been selected, the father is randomly chosen from all its conspecific individuals *l* in the community, with a probability proportional to their performance, *e*^*βC*_*l*_^. In other words, individuals with lower competition pressure are more likely to sire descendants both as females and as males. *Inheritance, admixture and variation of phenotypes–* The traits of each single offspring are a stochastic interpolation of those of both parents with the possibility of variation: $T_{\text{new}}^{k}=\eta T_{\text{mother}}^{k}+\left(1-\eta \right)T_{\text{father}}^{k}+\xi^{k}$, for *k* = 1,…,*n*, where *η* is a random variable (uniformly distributed in [0, 1]) allowing for different levels of admixture for each offspring, and *ξ*_*k*_ are (Gaussian) zero-mean random variables with standard deviation *μ*, a key parameter that characterizes the variability of inherited traits. To preserve the overall constraints *T*^*k*^ ∈ [0, 1] and ∑_*k*_ *T*^*k*^ = 1, mutations are generated as *ξ*^*k*^ = (*r*^*k*^ − *r*^*k*+1^), where {*r*^1^ = *r*^*n*+1^, …, *r*^*n*^} are independent Gaussian random variables with zero-mean and standard deviation $\mu /\sqrt{2}$; in the rare case that $T_{\text{new}}^{k}<0$ (resp. >1), we set it to 0 (resp. to 1) and added the truncated difference to another random trait.

### Biodiversity indices

*The centroid of species* *s* is **B**(*s*) = {*B*^1^(*s*), …, *B*^*n*^(*s*)}, with *B*^*k*^(*s*) = ∑_*i*_ *T*^*k*^(*i*)/*n*_*s*_ for each trait *k*, where *i* runs over the *n*_*s*_ individuals of species *s*. *Interspecies distance*: is the distance between the centroids of two different species *s* and *s*′ in the trade-off space *d*_*s*,*s*′_ = ∑_*k*_|*B*^*k*^(*s*) − *B*^*k*^(*s*′)|/*n*, averaged over all surviving species. *Intraspecific distance* is the average distance in trade-off space between all pairs of individuals of a given species *s*, *d*_*s*_ = ∑_*i*,*j* ∈ *s*_ *c*_*ij*_/*n*_*s*_(*n*_*s*_ − 1) averaged over all surviving species. *Local complementarity* is the mean complementarity of individuals to their spatial neighbors, LC = ∑_*i*_(∑_*j* ∈ *n*.*n*.(*i*)_ *c*_*ij*_/*K*)/*N* where *N* is the total number of individuals and *K* is the number of local neighbors. *Global complementarity* is the complementarity averaged over all pairs of individuals regardless of their relative positions in physical space, GC = ∑_*i*,*j* ≠ *i*_ *c*_*ij*_/(*N*(*N* − 1)). Similarly GC_inter_ is the averaged complementarity between individuals of different species and GC_*intra*_ is the averaged complementarity between conspecific individuals. In the case of monocultures, GC_inter_(*S* = 1) is measured from two different/independent realizations. *Relative complementarity*, RC = GC_inter_ − GC_intra_, is a measure of the averaged difference in the level of competition between randomly sampled conspecific and non-conspecific individuals. *Moran’s index*: is a measure of spatial correlations between neighbors; it is negative when neighbors tend to belong to different species (see S2 Appendix in S1 Text).

## Supporting Information

S1 Text **Here we provide several supplemental appendices**: *“S1. Local and global trait complementarities”* **provides three different measures: i) Local complementarity (LC), ii) Intraspecific global complementarity** (**GC**_intra_), **and, additionally, iii) Interspecies global complementarity** (**GC**_inter_); *“S2. Moran index”* **defines the Moran’s index**; *“S3. Long-range dispersal and competition”* **studies well mixed (or “fully connected”) communities**; *“S4. Comparison with neutral theory* (*β* = 0)” **analyses the limit of no competition *β* = 0 in which our model reduces to the neutral-theory**; *“S5. Initial phenotypic traits”* **studies a more realistic situation in which, at initial time, individual traits are distributed in the phenotypic space accordingly to its species**; *“S6. Emergent phenotypically equivalent species”* **studies the robustness of species coexistence during long periods of time and compares the mean time of coexistence between such species with expectations of neutral dynamics**; *“S7. Breach of symmetry”* **considers a model variant in which positions in the trade-off space are *not* equally rewarding a priori**; *“S8. Asexual reproduction”* **analyzes the case of asexual reproduction in which the traits are directly transmitted from an individual to its offspring (with some variability)**. *“S9. Choice of the competition kernel”* **shows the phenomenology for different competition kernels; and, finally**, *“S10. Surviving species”* **provides the number of surviving species in time**.
(PDF)
