# Supplementary material for: Eco-evolutionary Model of Rapid Phenotypic Diversification in Species-Rich Communities
Source: PLoS Comput Biol. 2016 Oct 13;12(10):e1005139. doi: 10.1371/journal.pcbi.1005139 (PMC5063285; doi:10.1371/journal.pcbi.1005139)
Supplement: S1 Text — Here we provide several supplemental appendices: “S1. Local and global trait complementarities” provides three different measures: i) Local complementarity (LC), ii) Intraspecific global complementarity (GCintra), and, additionally, iii) Interspecies global complementarity (GCinter); “S2. Moran index” defines the Moran’s index; “S3. Long-range dispersal and competition” studies well mixed (or “fully connected”) communities; “S4. Comparison with neutral theory (β = 0)” analyses the limit of no competition β = 0 in which our model reduces to the neutral-theory; “S5. Initial phenotypic traits” studies a more realistic situation in which, at initial time, individual traits are distributed in the phenotypic space accordingly to its species; “S6. Emergent phenotypically equivalent species” studies the robustness of species coexistence during long periods of time and compares the mean time of coexistence between such species with expectations of neutral dynamics; “S7. Breach of symmetry” considers a model variant in which positions in the trade-off space are not equally rewarding a priori; “S8. Asexual reproduction” analyzes the case of asexual reproduction in which the traits are directly transmitted from an individual to its offspring (with some variability). “S9. Choice of the competition kernel” shows the phenomenology for different competition kernels; and, finally, “S10. Surviving species” provides the number of surviving species in time. (PDF) [file pcbi.1005139.s001.pdf]

SUPPLEMENTARY INFORMATION
"Eco-evolutionary Model of Rapid Phenotypic Diversification in Species-Rich Communities."

P. Villa Martín,[1] J. Hidalgo,[1,2] R. Rubio de Casas,[3,4,5] and Miguel A. Muñoz[1*]

[1]Departamento de Electromagnetismo y Física de la Materia, and Instituto Carlos I de Física Teórica y Computacional, Universidad de Granada, 18071 Granada, Spain.

[2]Dipartimento di Fisica e Astronomia "Galileo Galilei", Università di Padova, 35131 Padova, Italy

[3]Estación Experimental de Zonas Áridas, EEZA-CSIC, Carretera de Sacramento s/n, Almería, Spain

[4]UMR 5175 CEFE - Centre d'Ecologie Fonctionnelle et Evolutive (CNRS), 1919 Route de Mende, F-34293 Montpellier cedex 05, France

[5]Departamento de Ecología, Universidad de Granada, Avda. de la Fuentenueva s/n, Granada 18071

[*]E-mail: mamunoz@onsager.ugr.es

Contents

S1 Local and global trait complementarities 2

S2 Moran index 3

S3 Long-distance dispersal and competition 4

S4 Comparison with neutral theory ($\beta = 0$) 5

S5 Initial phenotypic traits 6

S6 Effect of the competition kernel 7

S7 Emergent Neutrality 8

S8 Asymmetrical resource trade-offs 9

S9 Asexual reproduction 10

S10 Surviving species 11

S1 Local and global trait complementarities

We distinguish three different measures regarding averaged complementarities (see the sketches in Fig. S1): *i)* **Local complementarity (LC)**, defined as the mean phenotypic distance between an individual and its spatial neighbors; *ii)* **Intraspecific global complementarity (GC_{intra})**, defined as the mean phenotypic distance between all pairs of individuals within the same species (and then averaged over species); and, additionally, *iii)* **Interspecific global complementarity (GC_{inter})**, corresponding to the the mean phenotypic distance between all pairs of individuals consisting of individuals of two different species (averaged over all species). While local measurements capture the effect of spatial correlations, global ones are useful to characterize the evolution of the whole community in trade-off space. In analogy with the experimental setup of Zuppinger-Dingley *et al.* [1], we performed computer simulations using both monocultures and mixtures. For the case of monocultures, GC_{inter} was estimated taking individuals coming from two independent realizations of the simulations. Zuppinger et al. gathered seeds from surrounding populations and grew them together in their experimental set-up. This removed any cumulative, trans-generational effect of spatial correlations. In this way, GC measurements (Fig. S1 B) constitute better proxies (as compared to LC) to contrast our results with global community (biodiversity) effects in [1].

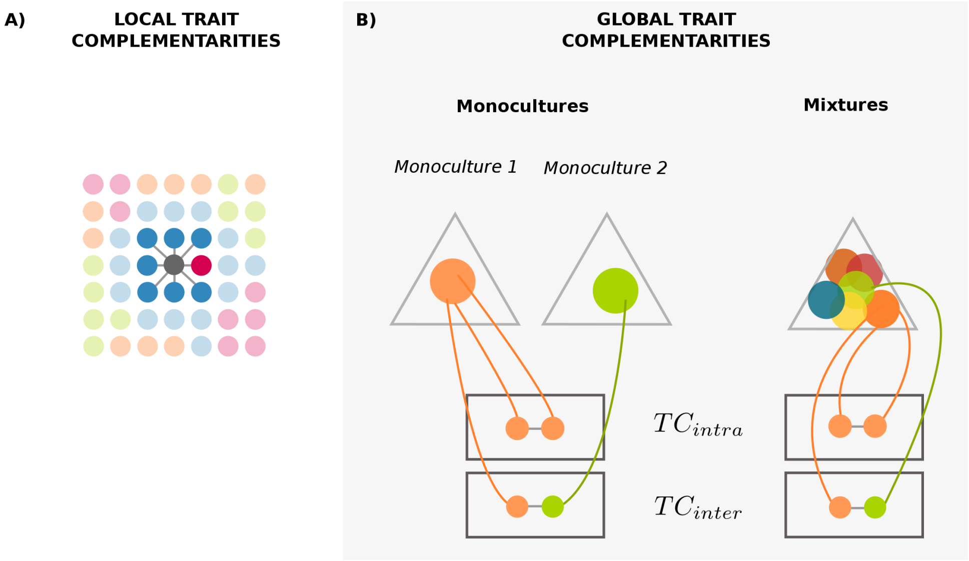

Supplementary Figure S1. (Color online) complementarity measurements: A) Local complementarity (i.e. mean phenotypic distance between spatially close neighbors) reveals the effect of spatial correlations; B) Intraspecific and interspecific global complementarities (i.e. mean phenotypic distance between individuals of the same and of different species, respectively, regardless of their spatial location). In all cases, we performed computer simulations using monocultures and mixtures.

Complementing the results presented in Fig. 3 of the main text, Fig. S2 shows measurements for LC and $\mathrm{GC}_{\mathrm{intra/inter}}$ for different initial number of species S after 10 and 1000 generations. We observe that, even though complementarities become almost independent of S at $t = 1000$ (due to the extinction of some species), transient measurements at $t = 10$ clearly show that communities with fewer species exhibit higher values of GC and, consequently, reach the stationary state faster. Biodiversity delays the process because several species simultaneously compete for empty niches. On the other hand, LC is inversely correlated with S, i.e., individuals tend to be more phenotypically similar to ás in less biodiverse communities.

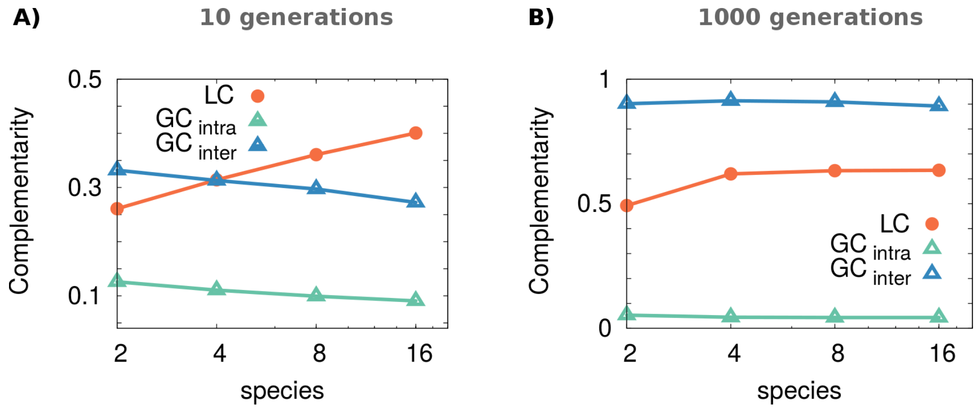

Supplementary Figure S2. (Color online) Local and global complementarities after A) 10 **and B)** 1000 **reproductive cycles**, plotted as a function of the initial number of species S in the community. Parameter values (see main text): system size $L = 64$, competition $\beta = 10$ and variability $\mu = 0.025$.

S2 Moran index

The Moran's index [2] quantifies the likelihood of an individual to be surrounded by individuals of the same species. When Moran's index is negative, individuals are less likely to be close to their co-specifics than what would be expected by pure chance, while positive values indicate spatial clustering of species. Mathematically, given a species s we compute its Moran's index I_s as

$$I_s = \frac{\sum_{i \in s} \sum_{j \in n.n.(i)} (X_s^i - \bar{X}_s)(X_s^j - \bar{X}_s)}{K \sum_{i \in s} (X_s^i - \bar{X}_s)^2}, \tag{1}$$

where K is the number of local neighbors (kernel size), and X_s^i is a variable such that $X_s^i = 1$ when the specie of i is equal to s and $X_s^i = 0$ if it is different, with $\bar{X}_s$ the density of individuals of species s. Finally, we obtain the total index averaging over species, $I = \sum_{i=1}^{S} I_s/S$. As a result, positive, zero, and negative values of I correspond to positive spatial correlation, random, and anti-correlation of species, respectively.

S3 Long-distance dispersal and competition

We have also studied well mixed (or “fully connected”) communities, in which *i)* there is frequent long-distance dispersal (so that both progenitors of the new offspring can be located at any site in space) and, additionally, *ii)* each individual competes with the rest of the community, i.e. all individuals behave as nearest neighbors.

As illustrated in Fig. S3, all the previously reported phenomenology is still present in this ideal mean-field scenario. As a matter of fact, phenotypic differentiation seems to occur faster than when spatial distribution is conditioned by local dispersal (see Fig. 3). In other words, long-distance dispersal and global competition drive evolution faster than local dynamics. This is a consequence of enhanced competition, which increases the relative fitness of better performing individuals. Another important difference is that, under mean field conditions, equivalent taxa cannot occupy different spatial locations and are forced to compete with each other. Consequently, coexistence of species with similar traits is much less likely than in spatially-explicit communities.

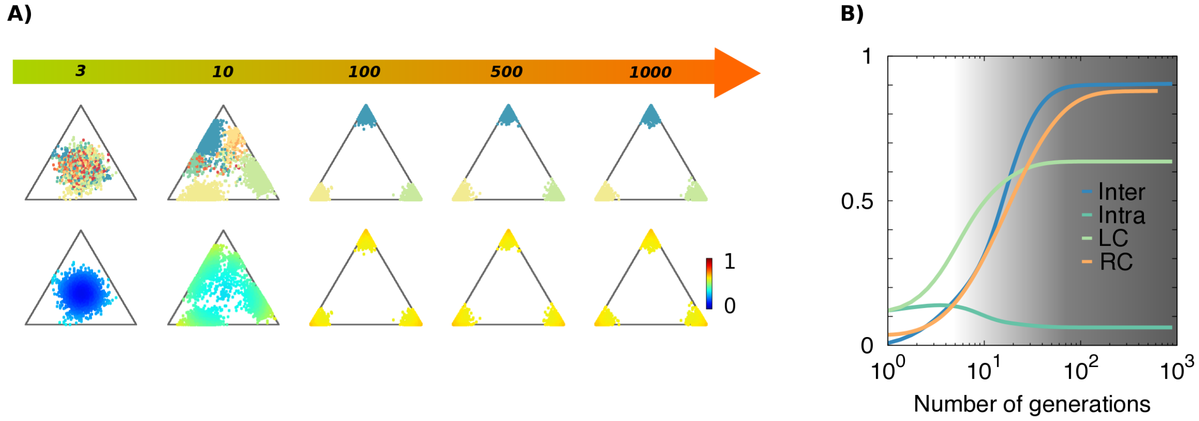

Supplementary Figure S3. (Color online) Evolution of the community with long-distance dispersal and global competition (i.e. well-mixed or mean-field dynamics). A) Tradeoff space and complementarity measured at different generations. B) Inter and intra-specific distances, and local complementarity and relative complementarity (RC= $\mathrm{GC_{inter}}$-$\mathrm{GC_{intra}}$) in time. Parameters: $L = 64$, $S = 16$, $\beta = 10$, $\mu = 0.025$.

S4 Comparison with neutral theory ($\beta = 0$)

In the limit of no competition, $\beta = 0$, our model equates to neutral-theory [3] in which reproduction probabilities become independent of individual phenotypes. Fig. S4 reports computational results for this case, illustrating the emergence of a very different scenario with respect to the non-neutral case. Sexual reproduction still pulls species together so they aggregate in the trade-off space, but their centroids describe slow and independent random walks instead of being controlled by a relatively fast separating drift (see Fig. S5). This phenomenology is caused by the lack of an effective force pushing species away; indeed segregated species can become closer after some generations, but on average there is only random drift allowing them to slowly diversify, so they cannot account for the empirical observations in [1]. Similarly, relative complementarities (which in the absence of competition can be regarded as the averaged difference in the level of phenotypic similarity between randomly sampled non-conspecific and conspecific individuals) start to grow later and reach low values,

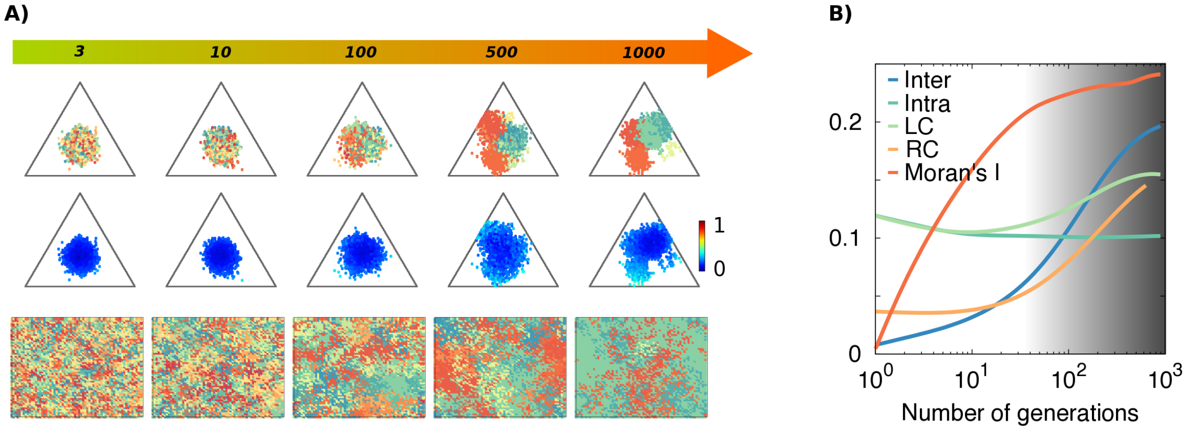

Supplementary Figure S4. (Color online) Neutral dynamics ($\beta = 0$), implying that all individuals have the same probability of reproduction independently of their species assignment and phenotype. A) Plots in the trade-off space illustrate that species hardly segregate in short time scales. In the physical space, local dispersal leads the the system to be clustered, i.e. positively auto-correlated rather than anti-correlated. B) Different measures illustrate that rapid evolutionary changes are much harder to observe in the neutral scenario. In particular, local complementarity (LC) and relative complementarity increase at a much slower pace. The increase in the Moran's index confirms that the system remains positively correlated (as usually is the case in neutral models) Parameters have been set to $L = 64$, $S = 16$ and $\mu = 0.025$.

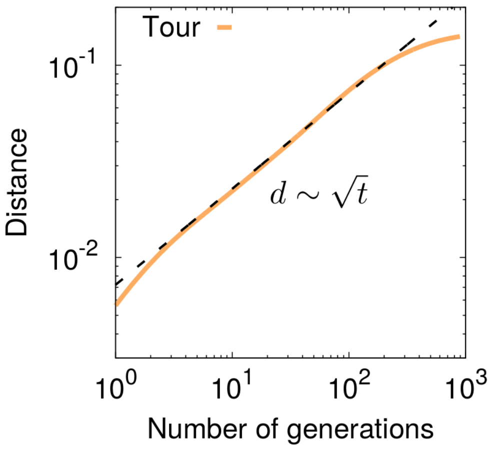

Supplementary Figure S5. (Color online) Species diffuse in the trade-off space under neutral dynamics ($\beta = 0$): The plot shows average distance between species centroids and the central point of the phenotypic space (i.e. the position of all species centroids at $t = 0$) as a function of time. Mutations cause a random movement of species centroids in the phenotypic space, as shown by the 0.5 slope in double logarithmic scale, characteristic of diffusive processes. Parameter values are set as in Fig. S5.

S5 Initial phenotypic traits

In the main text, initial conditions are given by randomly sampling the value of each individual phenotypic trait from a single distribution (Gaussian around the center of the phenotypic space), independently of species labels. After some generations, we observe that competition causes species to segregate in phenotypic space.

Although the most widely accepted definitions of species are based exclusively on the role of mating barriers, individuals belonging to the same species tend to share common trait values. In this appendix, we approximate this kind of scenario and test the robustness of our results running simulations with partial clustering of species in phenotypic space. For this, we sampled individual traits from equal amplitude Gaussian distributions centered around different (randomly chosen) species-dependent points of the phenotypic space (see initial top panel in Fig. S6).

As illustrated in Fig. S6, species diversify sooner in phenotypic space (initial values for inter- and intra-specific distances are higher than in the case described in the main text) but after this transient difference asymptotic results remain essentially unchanged.

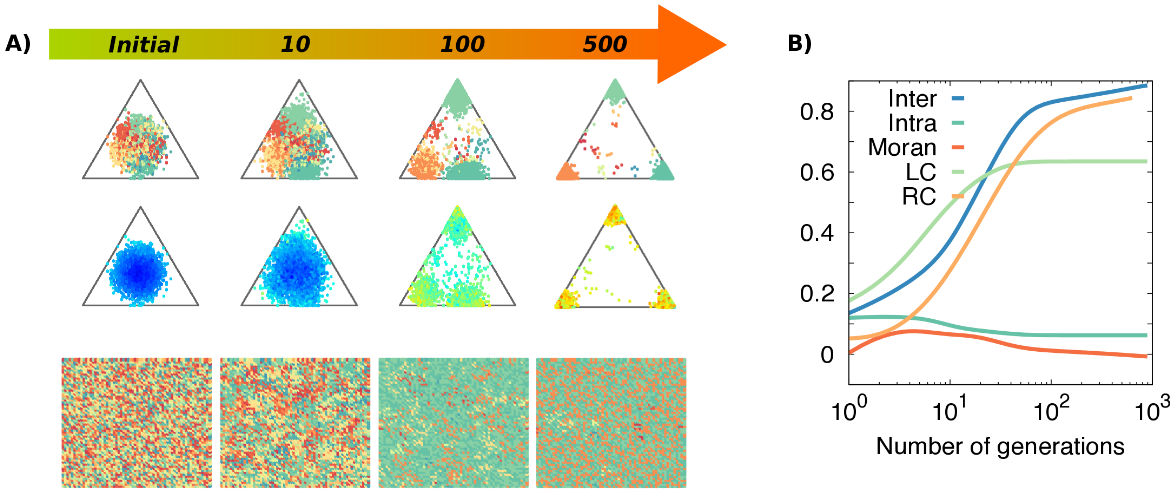

Supplementary Figure S6. (Color online) Simulations under initial phenotypic segregation of species. Individual traits are initially sampled from different species-dependent Gaussian distributions. Each of these Gaussians have a standard deviation equal to 0.05 and a mean value randomly selected from another Gaussian (with the same amplitude 0.05) centered at the triangle barycenter. A) From top to bottom: Tradeoff values, trait complementarities, and spatial distributions at different generations. B) Evolution of the inter and intra-specific distances, local and relative complementarity and Moran's Index. Parameters: $L = 64, S = 16, \beta = 10, \mu = 0.025$.

S6 Effect of the competition kernel

In the implementation of our model presented in the main text, the reproduction probability of an individual i is proportional to $e^{\beta C_i}$, where C_i is the average trait complementarity among neighbors, $C_i = \frac{1}{K}\sum_j \frac{1}{n}\sum^k |T^k(i) - T^k(j)|$. However, the use of a non-differentiable argument (absolute value) appearing linearly in the exponential kernel may lead to spurious robust coexistence of arbitrarily similar species (at zero phenotypic distance) [4, 5, 6, 7, 8]. Kernels of the form $e^{\beta C_i^{2+\alpha}}$ with $\alpha > 0$ have been shown to avoid such artifacts [9, 10]. For these reasons, we also considered an alternative competition kernel of the form $e^{\beta C_i^4}$ to check for the validity and robustness of our conclusions.

Results are shown in Fig S7. We observe that, as the quartic kernel reduces the overall competition of phenotypically similar individuals, it leads to a slower species-diversification process (as compared with the linear one for the same value of the parameters). However, results are qualitatively similar to the linear kernel case. In particular, similar (equivalent) species continue to emerge and coexiste for very long times, as in the linear case. In Appendix SS7 we discuss the coexistence of emergent equivalent species in more detail.

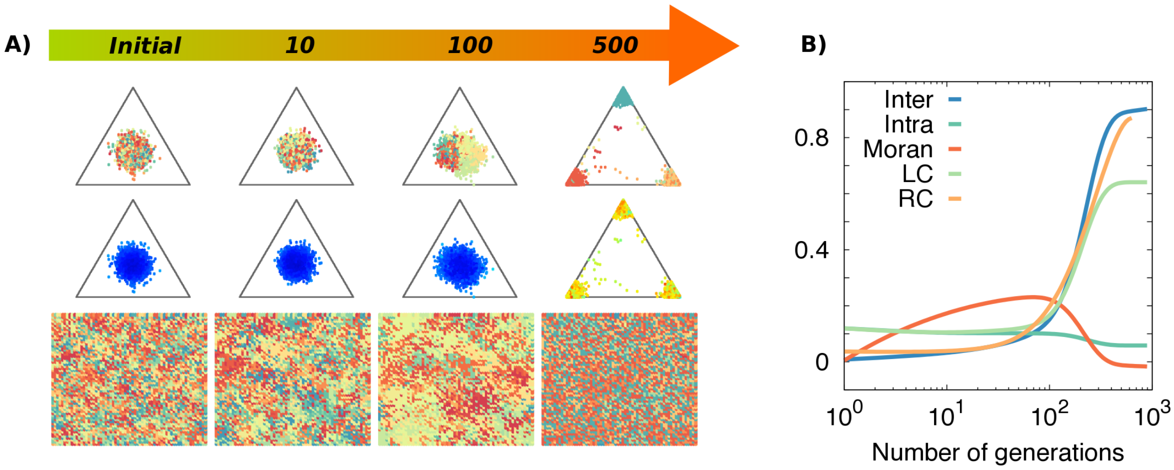

Supplementary Figure S7. (Color online) Quartic competition kernel. A) From top to bottom: Tradeoff values, trait complementarities, and spatial distributions at different generations. Simulations were run setting the performance of individuals proportional to $e^{\beta C^4}$ (rather than $e^{\beta C}$ used in the main text), where C is the average trait complementarity among neighbors. B) Inter and intra-specific distances, local and relative complementarity and Moran's Index evolution. Parameters: $L = 64, S = 16, \beta = 10, \mu = 0.025$.

S7 Emergent Neutrality

In the main text, we discuss the possibility of emerging phenotypically-equivalent species coexisting for long periods of time. It is important to underline that not all realizations lead to equivalent species coexisting in the community. However, this appears to be a significant pattern and we explored it further. In particular, we decided to check the stability of the coexistence. In this Section, we study the mean coexistence time of equivalent species as a measure of coexistence stability.

Two equivalent species coexist until one of them invades the phenotypic space of the other as a result of demographic fluctuations. As a first step to quantify the dynamics of this process, we define a computational criterion to determine equivalence: two species s_1 and s_2 are considered equivalent if their inter-specific distance (i.e. distance between their centroids) differs less than a fraction of their mean intraspecific distance (mean trait amplitude), for instance 1/4, which produces a significant overlap of the clusters of both species in phenotypic space.

We then measured the mean number of generations ΔT between the time at which 4 species remain in the system (with two of them being equivalent, based on the previous definition) and the time at which one of such equivalent species invades the other one. In voter models (i.e. the neutral case), the mean time to reach mono-dominance, ΔT, increases with the number of individuals in the community, N; in particular, $\Delta T \sim N \log N$ in a 2D lattice and $\Delta T \sim N$ in a well-mixed situation (for instance, the case of long-distance dispersal and global competition) [11].

Fig. S8 shows ΔT for simulations with limited dispersal (i.e. the 2D case), as well as the theoretical expectation for the neutral case. To check the robustness of coexistence to the shape of the competition kernel [4, 5, 9, 6, 10, 7, 8] (see Appendix SS6), we run simulations using a linear-exponential ($e^{\beta C}$) and a quartic-exponential ($e^{\beta C^4}$) kernel, where C is the mean trait complementarity among neighbors. In both cases, our results are compatible with the neutral scenario.

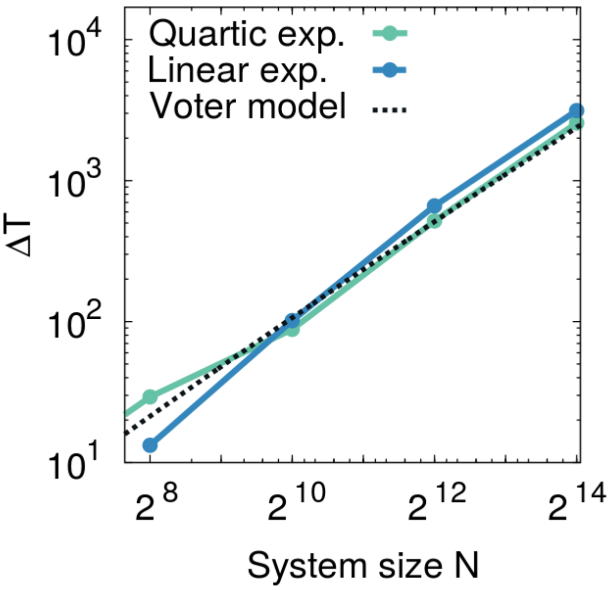

Supplementary Figure S8. (Color online) Mean number of " coexistence", for different system sizes of the community. We show results using a linear-exponential ($e^{\beta C}$) and a quartic-exponential ($e^{\beta C^4}$) kernel comparing them with the theoretical expectation for a 2D voter model, $\Delta T \propto N \log N$ [11]. Parameters: $L = 64, S = 8, \beta = 10, \mu = 0.025$. Deviations from the straight line probably stem from lack of statistics (which is costly at such large sizes/times).

S8 Asymmetrical resource trade-offs

In this section we consider a model variant in which positions in the trade-off space are *not* equally rewarding a priori. In particular, we chose one of the corners to be favored respect to the others: individuals whose phenotypes are closer to that vertex have a higher probability of reproduction. This could be interpreted as one particular limiting resource being more crucial, or mean that the availability of some resource scales nonlinearly with corresponding trait (e.g., a plant with a short root might not be able to reach a deep water layer. Individuals with longer root systems will have a disproportionate advantage).

In particular, we now modulate the performance of each individual i by multiplying it by a factor $R_i = r_1 T^1 + r_2 T^2 + r_3 T^3$; where r_1, r_2 and r_3 are weights (real numbers in the interval $[0, 1]$) such that $r_1 + r_2 + r_3 = 1$. For simplicity we fix $r_1 = (1 + 2\epsilon)/3$, $r_2 = r_3 = (1 - \epsilon)/3$ in order to control the asymmetry with a single parameter (ϵ; the symmetric case is $\epsilon = 0$), while ϵ values close to 1 lead to large asymmetries. In what follows we fix $\epsilon = 0.99$.

As illustrated in Fig. S9, although most of individuals initially occupy the most favored (left) corner, after a few generations, some individuals also settle at other available (and less favorable) regions; this is a consequence of the system's tendency to reduce the level of competition. In conclusion, the main phenomenology reported in the main text appears robust to asymmetrical trade-offs.

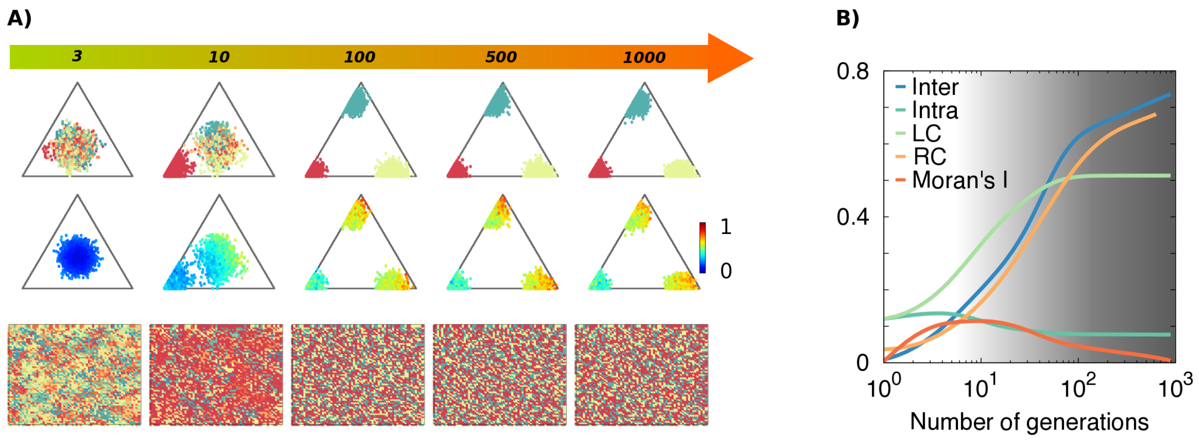

Supplementary Figure S9. (Color online) Asymmetry among trade-offs: A) Trade-off space and complementarity as a function of the number of generations. B) Different measurements characterizing the community in time. Local and relative complementarities confirm that rapid evolutionary changes may within a few generations. Parameters: $L = 64$, $S = 16$, $\beta = 10$, $\mu = 0.025$.

S9 Asexual reproduction

Our model adopts the (sexual) reproduction mechanism of the communities considered in the experiments by Zuppinger-Dingley *et al.* [1]. Here we analyze a case of asexual reproduction in which the traits are directly transmitted from an individual to its offspring (with some variability), i.e. taking $\eta = 1$ in our model (see main text).

Fig S10A shows the evolution of individual phenotypes (each trait value T_1, T_2, T_3 is represented by the amount of red, yellow and blue respectively), complementarity and spatial distribution. Observe that, once again, the chief phenomenology of the model, i.e. segregation toward high levels of specialization, is observed. However, in this case, the mechanism of diversification is quite different: individuals from any given species can specialize independently. Thus, in the absence of sexual reproduction, diversification occurs at an individual rather than at a species level. This type of individual differentiation fosters the presence of equivalent species (as the species label becomes completely irrelevant in this setting).

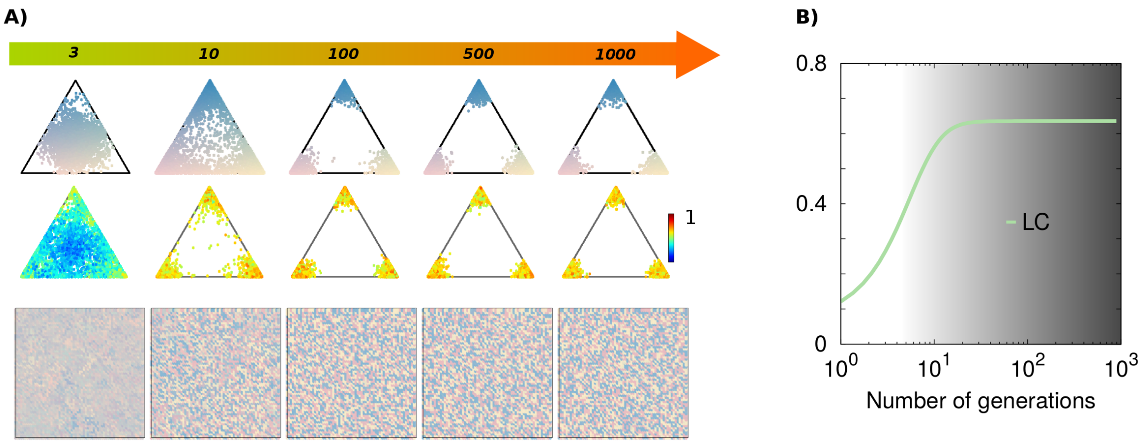

Supplementary Figure S10. (Color online) Asexual reproduction: A) Tradeoff space and complementarity as a function of the number of generations. Each individual traits values T_1, T_2, T_3 are represented by the amount of red, yellow and blue respectively. Parameter values: $L = 64$, $S = 16$, $\beta = 10$, $\mu = 0.025$. Competition avoidance leads individuals to segregate in the trade-off space. B) Local complementarity increases similarly to the main model.

S10 Surviving species

As we do not include mechanisms such as migration or speciation, the number of species actually present in the community can be reduced after several generations. The resulting change in diversity can be regarded as an important attribute, because it illustrates the limit of maximum diversity that a finite system can harbor is the absence of inmigration or speciation processes.

Fig. S11 shows the number of surviving species in different scenarios, including different levels of competition and variability parameters, and other model variants. As expected, extinctions occur more rapidly for higher levels of competition (larger values of β). The effective level of competition is enhanced in the mean-field case in which all individuals interact with each other (see Appendix SS3), leading to faster extinction.

In the neutral case ($\beta = 0$), species disappear at a very slow rate as there is no competition, but due to stochasticity, most of them are likely to disappear, leading to mono-dominance for sufficiently large timescales [12]. Interestingly, the stable solution in our model with competition ($\beta \neq 0$) consists of multiple species –as many as the niche dimensionality, in this case 3– coexisting for an arbitrarily large number generations. This result is congruent with the “niche dimension hypothesis”, which states that a greater diversity of niches leads to a greater diversity of species [13].

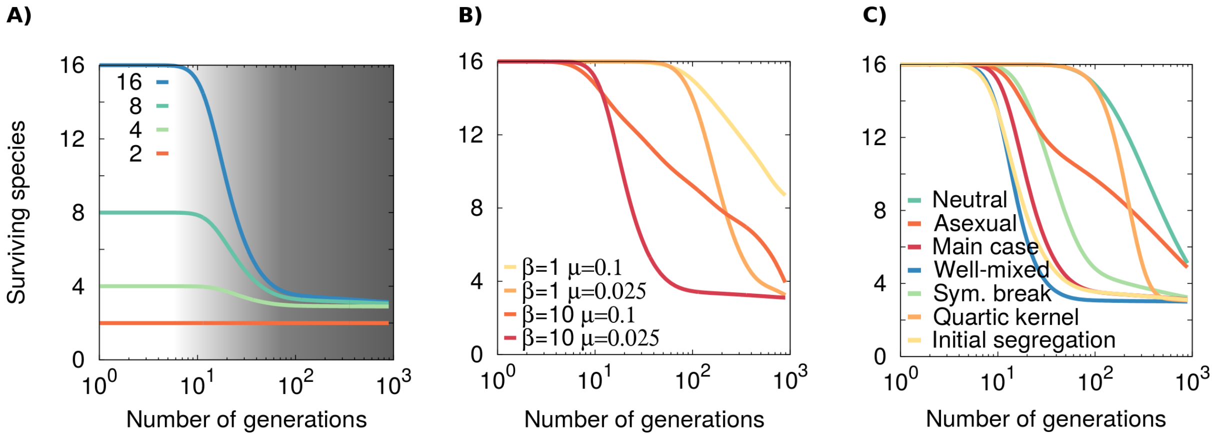

Supplementary Figure S11. (Color online) Number of surviving species in time for different A) initial number of species, B) competition and variability parameters and C) variants of the model. Species disappear faster in environments with high competition (higher values of β, or the mean-field). In contrast, the neutral case ($\beta = 0$) corresponds to the case in which more species survive after generations, although, due to demographic fluctuations, they still disappear on the long term. For sufficiently large numbers of generations, the system converges to a state with the same number of species than the niche dimensionality (3 in our case); these species coexist for arbitrarily long periods (provided the lattice is sufficiently large). Parameter values: $L = 64$, and $\beta = 10$, and $\mu = 0.025$ in A) and C).

References

[1] Zuppinger-Dingley D, et al. (2014) Selection for niche differentiation in plant communities increases biodiversity effects. *Nature* 515:108–111.

[2] Moran PA (1950) Notes on continuous stochastic phenomena. *Biometrika* pp 17–23.

[3] Hubbell SP (2001) *The unified neutral theory of biodiversity and biogeography (MPB-32)* (Princeton University Press) Vol. 32.

[4] Geritz SA, van der Meijden E, Metz JA (1999) Evolutionary dynamics of seed size and seedling competitive ability. *Theoretical population biology* 55:324–343.

[5] Adler FR, Mosquera J (2000) Is space necessary? interference competition and limits to biodiversity. *Ecology* 81:3226–3232.

[6] Hernández-García E, López C, Pigolotti S, Andersen KH (2009) Species competition: coexistence, exclusion and clustering. *Philosophical Transactions of the Royal Society of London A: Mathematical, Physical and Engineering Sciences* 367:3183–3195.

[7] Barabás G, D'Andrea R, Ostling AM (2013) Species packing in nonsmooth competition models. *Theoretical ecology* 6:1–19.

[8] Lampert A, Hastings A (2014) Sharp changes in resource availability may induce spatial nearly periodic population abundances. *Ecological Complexity* 19:80–83.

[9] Pigolotti S, López C, Hernández-García E (2007) Species clustering in competitive lotka-volterra models. *Physical review letters* 98:258101.

[10] Pigolotti S, López C, Hernández-García E, Andersen KH (2010) How gaussian competition leads to lumpy or uniform species distributions. *Theoretical Ecology* 3:89–96.

[11] Cox JT (1989) Coalescing random walks and voter model consensus times on the torus in zd. *The Annals of Probability* pp 1333–1366.

[12] Liggett T (2012) *Interacting particle systems* (Springer Science & Business Media) Vol. 276.

[13] Hutchinson G (1957) Concluding remarks. *Cold Spring Harbor Symposia on Quantitative Biology* 22:415–427.
